# Emerging SARS-CoV-2 variants follow a historical pattern recorded in outgroups infecting non-human hosts

**DOI:** 10.1038/s42003-021-02663-4

**Published:** 2021-09-22

**Authors:** Kazutaka Katoh, Daron M. Standley

**Affiliations:** Research Institute for Microbial Diseases, Osaka University, 3-1 Yamadaoka, Suita, 565-0871 Japan

**Keywords:** Virus-host interactions, Molecular evolution

## Abstract

The ability to predict emerging variants of SARS-CoV-2 would be of enormous value, as it would enable proactive design of vaccines in advance of such emergence. We estimated diversity of each site on a multiple sequence alignment (MSA) of the Spike (S) proteins from close relatives of SARS-CoV-2 that infected bat and pangolin before the pandemic. Then we compared the locations of high diversity sites in this MSA and those of mutations found in multiple emerging lineages of human-infecting SARS-CoV-2. This comparison revealed a significant correspondence, which suggests that a limited number of sites in this protein are repeatedly substituted in different lineages of this group of viruses. It follows, therefore, that the sites of future emerging mutations in SARS-CoV-2 can be predicted by analyzing their relatives (outgroups) that have infected non-human hosts. We discuss a possible evolutionary basis for these substitutions and provide a list of frequently substituted sites that potentially include future emerging variants in SARS-CoV-2.

## Abstract (web summary)

In order to improve our ability to predict emerging variants of SARS-CoV-2, Katoh and Standley analysed the molecular evolution of the Spike protein. They found a significant correspondence in the location of mutations between recently emerging SARS-CoV-2 variants and their relatives that infected bat and pangolin before the pandemic, which could suggest that the sites of future mutations could be predicted by analyzing their relatives that have infected non-human hosts.

## Introduction

In December 2020, three SARS-CoV-2 variants emerged with increased infectivity from England, South Africa, and Brazil. The fact that certain mutations in the Spike (S) protein had occurred independently prompted us to reexamine our September 2020 study of the evolution of this protein^1^. In our original study, we characterized the *importance* of each residue position in the S protein by comparing its diversity in SARS-CoV-2 with that in relatives (outgroups) that infected bats or pangolins by using a simple equation:

1 $${{Importance}}={{diversity}}({{{{{{{\rm{SARS}}}}}}}}{{{{{\mbox{-}}}}}}{{{{{{{\rm{CoV}}}}}}}}{{{{{\mbox{-}}}}}}2+{{{{{{{\rm{outgroup}}}}}}}})-{{diversity}}({{{{{{{\rm{SARS}}}}}}}}{{{{{\mbox{-}}}}}}{{{{{{{\rm{CoV}}}}}}}}{{{{{\mbox{-}}}}}}2),$$

where *diversity*(*x*) is defined as the number of different amino acids observed at the site in question in virus group *x*. This equation, which was meant to be descriptive rather than predictive, identified twenty positions of high *importance*. We were thus surprised to find that, of these 20 positions, four were characteristic of the above emerging variants: Histidine 69, Valine 70, Glutamine 484, and Asparagine 501. These sites coincide with four out of the five residues (69, 70, 417, 484, 501) that have mutated independently in two or more of the three emerging lineages or a lineage transmitted between human and mink^2^. We reanalyzed the underlying sequence data and found that the *importance* values of these sites were determined primarily by *diversity*(outgroup), rather than *diversity*(SARS-CoV-2). In hindsight, this is somewhat expected, as the latter term was close to unity at the time when we performed the analysis (i.e., before the emergence of new variants).

A natural question, then, is why a limited set of sites with high diversity in outgroups have also recently been substituted in SARS-CoV-2. As an evolutionary mechanism behind such frequent substitutions, two extreme scenarios, (i) neutral evolution and (ii) positive selection, are possible. These two scenarios give opposite predictions as to functionality of frequently substituted residues: scenario (i) predicts that the frequently substituted sites are not functionally important because they are under low functional constraints, while scenario (ii) predicts that functionally important sites have changed by being positively selected. Although the truth may lie in between these two extremes, we tested which scenario is more likely using the distribution of residues that are known to be important for infection to host cells.

## Results and discussion

### Known functionally important sites

**Fig. 1:**
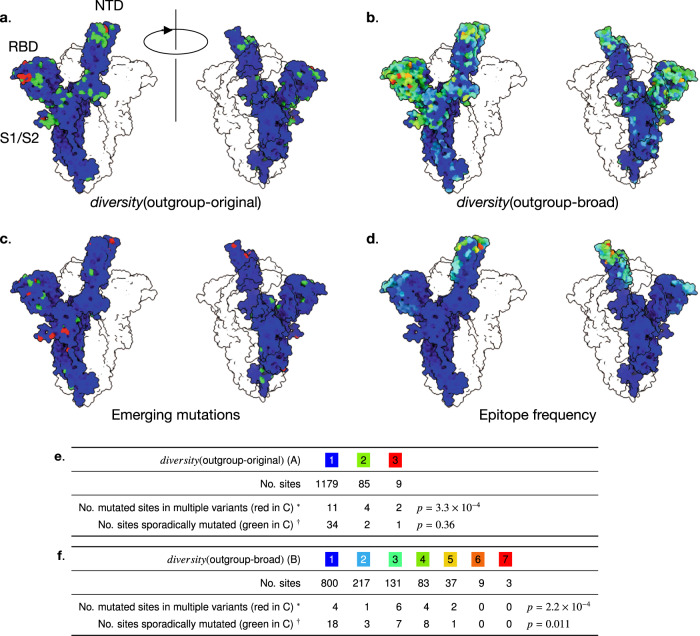
Diversity and other indices mapped on structure of the S protein, visualized by ChimeraX^28^. A movie of the structure rotating is available at https://mafft.cbrc.jp/alignment/pub/sarscov2/structure.mp4. For clarity, a single S protein is shown in the context of a spike trimer. **a**, **b** *diversity*(outgroup), the number of different amino acids observed at each site in outgroup, was computed using the original and broad definitions of outgroup: Low diversity (blue); High diversity (red). **c** Emerging mutations found in single variant (green); in multiple variants (red) infecting humans. **d** Epitope frequency, the number of antibodies that contact each residue ( <6 Å), was counted based on currently available Protein DataBank (PDB) entries of S protein-antibody complexes listed in https://mafft.cbrc.jp/alignment/pub/sarscov2/epitopefrequency.txt. 0 (blue); 15 (red). This value is not expected to represent all spike-targeting antibodies. **e** Correspondence between *diversity*(outgroup-original) (**a**) and emerging mutations (**c**). **f** Correspondence between *diversity*(outgroup-broad) (**b**) and emerging mutations (**c**). Positions of emerging mutations in **c**, **e**, and **f** were taken from reference^27^. Asterisk represents mutated sites in multiple variants = residues in bold in Table 2 in Peacock et al.^27^; dagger represents sporadic mutations = the other residues listed in the same table.

Currently available functional information supports scenario (ii). When viewing the distribution of residues with high *diversity*(outgroup) as a heatmap on the spike molecular surface (Fig. 1a, b), it is apparent that these residues are not evenly distributed, but form clusters in the N terminal domain (NTD), receptor binding domain (RBD) and S1/S2 cleavage site, which are thought to be important for interaction to human cells. More specifically, Glutamine 484 and Asparagine 501 are structurally close to the interface with the host cell receptor ACE2, which, in turn, is targeted by neutralizing antibodies. Histidine 69 and Valine 70, on the other hand, are far from the ACE2 binding site but proximal to a recently-reported epitope for infection-enhancing antibodies^3,4^. The 69/70 deletion mutant also occurred in an immunosuppressed individual who underwent convalescent plasma therapy^5^, suggesting that the mutation is a direct response to host antibodies. These two residues have also been reported to bind sialic acids^6^. There are also high diversity sites (around Alanine 684) adjacent to the S1/S2 cleavage site of SARS-CoV-2, as indicated in Table 1. The changes in this region seem to be host specific: HSMSS[LF]R in pangolin; QTQTNSR in two lineages of bat; QTQTNSPRRAR (which includes a polybasic insertion recognized by host’s protease^7,8^) in human. These changes might reflect adaptation to new hosts in the past. Further substitutions, such as Proline 681, could change infectivity in human^9^ around this region.

**Table 1:** High diversity residues.

Res	AA	Orig	Broad	Epitope	ACE2	SialicAcid	Cleavage	Emerge
8	L	1	5	0	–	–	–	–
9	P	1	5	0	–	–	–	–
12	S	1	5	0	–	–	–	–
22	T	1	5	0	–	–	–	–
23	Q	2*	6	0	–	o	–	–
24	L	2	5	0	–	o	–	–
25	P	2	5	0	–	–	–	–
27	A	2	7	0	–	–	–	–
66	H	2	5	0	–	–	–	–
69	H	3*	2	2	–	o	–	m
70	V	3 *	4	2	–	o	–	m
71	S	3 *	7	1	–	–	–	–
72	G	2 *	6	1	–	–	–	–
73	T	3 *	4	1	–	–	–	–
74	N	3 *	5	1	–	–	–	–
76	T	3 *	7	2	–	–	–	–
85	P	1	5	0	–	–	–	–
137	N	2	6	0	–	–	–	–
140	F	1	5	0	–	–	–	–
147	K	2	5	4	–	–	–	–
164	N	1	5	0	–	–	–	–
169	E	1	5	0	–	–	–	–
176	L	1	6	0	–	–	–	–
183	Q	1	6	1	–	–	–	–
197	I	2	6	0	–	–	–	–
215	D	1	5	2	–	–	–	s
218	Q	2 *	5	2	–	–	–	–
224	E	1	5	0	–	–	–	–
249	L	1	5	3	–	–	–	–
253	D	3 *	4	2	–	–	–	s
255	S	2 *	4	1	–	–	–	–
256	S	1 *	3	0	–	–	–	–
260	A	2	5	1	–	–	–	–
272	P	2	5	0	–	–	–	–
324	E	2	5	0	–	–	–	–
417	K	2 *	3	8	o	–	–	m
439	N	2 *	6	0	–	–	–	–
440	N	2	5	0	–	–	–	–
441	L	2 *	3	3	–	–	–	–
443	S	1	5	1	–	–	–	–
444	K	2 *	5	0	–	–	–	–
445	V	2 *	4	3	–	–	–	–
449	Y	3	3	2	o	–	–	–
450	N	2 *	5	11	–	–	–	–
459	S	2	5	6	–	–	–	–
484	E	2	5	7	–	–	–	m
493	Q	2	5	10	o	–	–	–
501	N	2 *	5	8	o	–	–	m
504	G	2	5	8	–	–	–	–
529	K	2 *	3	0	–	–	–	–
532	N	2	5	0	–	–	–	–
554	E	2 *	4	0	–	–	–	–
556	N	2	5	0	–	–	–	–
640	S	2	5	0	–	–	–	–
677	Q	2 *	4	0	–	–	–	m
678	T	2	5	0	–	–	–	–
679	N	2 *	5	0	–	–	–	–
680	S	2 *	4	0	–	–	–	–
684	A	3	1	0	–	–	o	–
688	A	2	5	0	–	–	–	–
689	S	2 *	4	0	–	–	–	–

The most diverse residue positions are listed along with several annotations.

Orig, *diversity*(outgroup-original); Asterisk, (nonsynonymous substitutions)/(synonymous substitutions) > 1 in outgroup-original; Broad, *diversity*(outgroup-broad); Epitope, epitope frequency.

(See the caption of Fig. 1); ACE2, residue is within 6Å of ACE2 in PDB entry 7DF4; SialicAcid, reported sialic acid binding residue^6^; Cleavage, known protease cleavage site; Emerge, emerging variants in humans listed in Table 2 in Peacock et al. (2021)^27^. m, found in multiple variants; s, found in a single variant. See https://mafft.cbrc.jp/alignment/pub/sarscov2/fulllist.tsv for a full list.

### Possible positive selection in SARS-CoV-2 and outgroups

Modification of the regions discussed above could thus affect the infectivity or enable the virus to escape from the host’s immune system, albeit temporarily, as the change will inevitably be counteracted by a shift in the antibody repertoire of the host, resulting in an effective “arms race”, as reviewed in references^10,11^. In this scenario, the sites with higher diversity imply direct or indirect host–pathogen interactions and are thus in a constant state of flux. This interpretation is consistent with previous studies that reported the possibility of adaptive evolution to infect human in SARS-CoV-2^12,13^ and in other coronaviruses^14^. More recently, reports suggest that mutations in the B.1.1.7/B.1.351/P.1 lineages result in reduced binding affinity to some but not all neutralizing antibodies^15,16^. For close outgroups of SARS-CoV-2, where functional information in non-human hosts is not available, we explored the possibility of positive selection using Bayes Empirical Bayes analysis^17^ implemented in the PAML program^18^, excluding human-infecting lineages. The fourth column in https://mafft.cbrc.jp/alignment/pub/sarscov2/fulllist.tsv shows the sites estimated to have had more nonsynonymous substitutions than synonymous substitutions in the outgroup sequences, although this estimation is sensitive to sequence selection and alignment ambiguity.

### Correspondence of high diversity sites between SARS-CoV-2 and outgroups

Assuming adaptive evolution, it is conceivable that different sites are positively selected in different hosts to “optimize” infectivity; however, the analysis of outgroups revealed that such sites can overlap, presumably being involved in a common mechanism of host-pathogen interaction in different lineages, and that frequent changes in these sites already occurred in outgroups before the pandemic. We note that the correspondence between the positions of emerging mutations found in multiple human-infecting variants and those with high *diversity*(outgroup) is significant by Fisher’s exact test, regardless whether the original outgroup (Fig. 1a) or a broad outgroup (Fig. 1b) is used (see the lines marked with asterisk, *, in Fig. 1e, f). By contrast, the positions of sporadic mutations that are found just in a single variant in human show less clear or no correspondence with *diversity*(outgroup) (see the lines marked with dagger, †, in Fig. 1e, f). The former type of mutations (found in multiple variants) are likely to affect interactions with host factors and to spread in humans, although it’s difficult to differentiate between parallel evolution and recombination between lineages. The proposed simple method is suitable to predict such sites because they appear to be under positive selection in independent lineages including outgroups. There are some sites that have high diversity in outgroup but are not (yet) mutated in the current population of SARS-CoV-2. Such sites are regarded to be mis-predicted in this statistical test, but may mutate in the future. Indeed, residues close to the S1/S2 cleavage site were found to have high *diversity*(outgroup) in our initial analysis before emergence of several variants of concern (red sites in Fig. 1a) in 2020. Subsequently, substitutions in this region were indeed found in multiple variants infecting humans (red sites in Fig. 1c).

### Prediction of position of emerging mutations

To anticipate new variants of SARS-CoV-2 as early as possible, a straightforward strategy would be to intensively collect a large amount of sequence data from human-infecting lineages^19^. Our observation above leads to a complementary strategy: prepare against new variants in advance by decoding the long history of host-pathogen interactions recorded in the outgroup sequences infecting non-human hosts. Unfortunately, currently efforts have focused almost exclusively on the former strategy and available outgroup sequences are limited. If richer sequence data of outgroups infecting bat, pangolin and other possible hosts becomes available, it would not only shed light on the origin of SARS-CoV-2^20^, but also give us an advantage in the arms race with this virus.

### Limitations

**Fig. 2:**
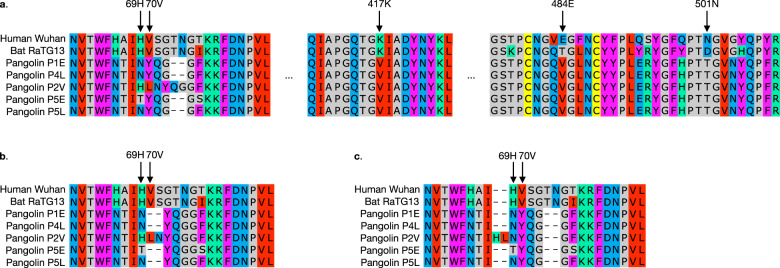
MSA around residues 69, 70, 417, 484, and 501, visualized by Jalview^29^. **a** MSA used in reference^1^. **b**, **c** Alternative MSAs around residues 69 and 70.

This analysis has several limitations. First, genetic changes can be caused by recombinations, not only point mutations and insertions/deletions. Indeed the receptor binding motif of SARS-CoV-2 was reported to be acquired from a lineage infecting pangolin^21^. It is possible that some changes in outgroups are also caused by recombinations. Our analysis regards a recombination simply as simultaneous changes in successive sites in the recipient genome. As a result, in comparison with the number of evolutionary events, diversity is overestimated if recombination between lineages occurred and the donor is not included in the alignment, while diversity is underestimated when the donor is included in the alignment. Second, the diversity in outgroup can be calculated only when the corresponding part exists in outgroups. This method cannot be applied to human-specific insertions. Third, the prediction of the position of a mutation can be ambiguous because of alignment ambiguity. Figure 2a shows the multiple sequence alignment (MSA) used in Saputri et al.^1^, and Fig. 2b shows an alternative MSA. Since the insertion in the P2V strain in pangolin should be independent from the insertion at human and RaTG13, gaps can be inserted in different positions between these two groups. Thus some other MSAs (eg, Fig. 2c) are also possible. By using different MSAs, the position of high diversity sites can shift. Even in this case, a high diversity region should exist nearby, because the alignment ambiguity itself is due to high diversity. This problem more frequently occurs when including a wider range of outgroups. Thus, to obtain a prediction at the residue-level resolution, a large amount of data from close outgroups is necessary.

## Methods

### Sequence data to calculate diversity

According to the interpretation of positive selection resulting in an “arms race”, it is possible that positions of mutations in future emerging variants can be predicted simply by identifying sites with high diversity in outgroups, where adversarial host-pathogen interactions have been occurring longer than for SARS-CoV-2 and humans. Because of their potential importance in the design of vaccines against future emerging variants, we calculated *diversity*(outgroup) for each residue position considering two definitions of outgroups: one that is identical to that used in our original analysis in which 6 sequences were used and a broader definition (18 sequences) to increase the amount of data used in the calculation. Both datasets are available at https://mafft.cbrc.jp/alignment/pub/sarscov2/. The sequence data was taken from GISAID^22^ and genbank, to cover major lineages of outgroups appearing in recent reports^23,24^. As noted in Introduction, *diversity*(*x*) is defined as the number of different amino acids observed at the site in question, where *x* is either of the two outgroups. Amino acid residues with high *diversity*(outgroup) are listed in Table 1.

### Tree

**Fig. 3:**
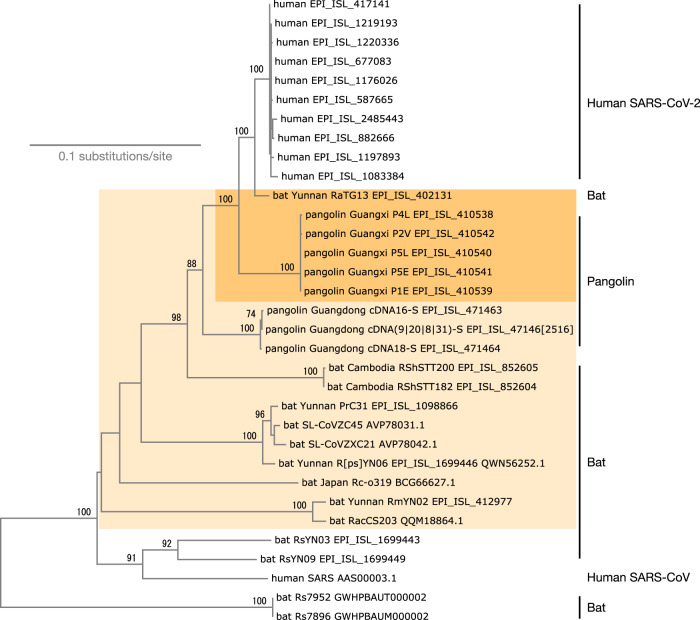
Outgroup sequences used as “original” (dark orange) and “broad” (light orange), displayed on a simple tree. Bootstrap values larger than 70% Accession number in GISAID, genbank or National Genomics Data Center is given for each sequence.

Figure 3 shows a phylogenetic tree of the S protein from SARS-CoV-2 and relatives including remote ones by the neighbor-joining method^25^ applied to a distance matrix estimated with the Poisson correction based on an amino acid sequence alignment by MAFFT^26^. The outgroup sequences used here are highlighted in this figure. The accession numbers of the sequences are given in this tree.

### Statistics and reproducibility

In Fig. 1e, f, *p* values were calculated by Fisher’s exact test under the null hypothesis that the diversity of each site in outgroups (Fig. 1a or b) and the distribution of emerging mutations (Fig. 1c) are independent of each other. Positions of the mutations found in multiple variants and the sporadic mutations in Fig. 1c were taken from Peacock et al.^27^.

### Reporting summary

Further information on research design is available in the Nature Research Reporting Summary linked to this article.

## Supplementary information

Reporting Summary

## Data Availability

Sequence data used here are available at https://mafft.cbrc.jp/alignment/pub/sarscov2/.

## Code availability

A script to count the number of amino acids in each site is available at https://mafft.cbrc.jp/alignment/pub/sarscov2/.
